# PHINDaccess Hackathons for COVID-19 and Host-Pathogen Interaction: Lessons Learned and Recommendations for Low- and Middle-Income Countries

**DOI:** 10.1155/2023/6638714

**Published:** 2023-10-10

**Authors:** Kais Ghedira, Hamza Dallali, Monia Ardhaoui, Zied Bouslema, Yosr Hamdi, Salma Feki Ben-Salah, Hanen Chelbi, Chiraz Atri, Melek Chaouch, Naira Dekhil, Afef Rais, Saifeddine Azouz, Manel Gharbi, Fatma Guerfali, Chaima Hkimi, Selim Kamoun, Ayoub Ksouri, Imen Moumni, Houyem Ouragini, Raghda Bsibes, Zeineb Afifi, Khouloud Youssfi, Hichem Ben Hassine, Najet Hadhri, Helmi Mardassi, Houcemeddine Othman, Oussema Khamessi

**Affiliations:** ^1^Laboratory of Bioinformatics, Biomathematics and Biostatistics LR20IPT09, Pasteur Institute of Tunis, University of Tunis El Manar, Tunis 1002, Tunisia; ^2^Laboratory of Biomedical Genomics and Oncogenetics (LR20IPT05), Pasteur Institute of Tunis, University of Tunis El Manar, Tunis 1002, Tunisia; ^3^Department of Human and Experimentally Anatomic Pathology, Laboratory of Molecular Epidemiology and Experimental Pathology, Institut Pasteur de Tunis, University of Tunis El Manar, Tunis, Tunisia; ^4^Laboratory of Molecular Epidemiology and Experimental Pathology, Tunisia; ^5^Laboratory for Rabies Diagnostics, Institute Pasteur of Tunis, Belvedere, Tunis 1002, Tunisia; ^6^University of Tunis El Manar, Tunis, Tunisia; ^7^Laboratory of Virus, Vector and Hosts (LR20IPT02), Institut Pasteur de Tunis, Université Tunis El Manar, Tunis 1068, Tunisia; ^8^Laboratory of Medical Parasitology, Biotechnology and Biomolecules, LR16IPT06, Institut Pasteur de Tunis, Université Tunis El Manar, Tunis Belvédère 1002, Tunisia; ^9^Laboratory of Transmission, Control and Immunobiology of Infections (LTCII), LR16IPT02, Institut Pasteur de Tunis, Université Tunis El Manar, Tunis Belvédère 1002, Tunisia; ^10^Laboratory of Molecular Microbiology, Vaccinology, And Biotechnology Development, Institut Pasteur de Tunis, Université Tunis El Manar, Tunis, Tunisia; ^11^Genomics Platform, Institut Pasteur de Tunis, Université Tunis El Manar, Tunis 1068, Tunisia; ^12^Laboratory of Epidemiology and Veterinary Microbiology. Group of Bacteriology and Biotechnology Institut Pasteur of Tunisia, University of Tunis El Manar (UTM), Tunis 1002, Tunisia; ^13^Laboratory of Venom, Toxins and Therapeutic Molecules, Institut Pasteur Tunis, University Tunis El Manar, Tunis, Tunisia; ^14^Laboratory of Molecular and Cellular Hematology, LR16IPT07, Pasteur Institute of Tunis, University of Tunis El Manar, Tunisia; ^15^Grant Office, Institut Pasteur de Tunis, Tunis, Tunisia; ^16^Specialized Unit “Communication, Science and Society”, Institut Pasteur de Tunis, Tunis, Tunisia; ^17^Sydney Brenner Institute for Molecular Bioscience, Faculty of Health Sciences, University of the Witwatersrand, Johannesburg, South Africa; ^18^Department of Genetics, Farhat Hached University Hospital, Sousse, Tunisia; ^19^Laboratory of Cytogenetics, Molecular Genetics, and Reproductive Biology (LR03SP02), Farhat Hached University Hospital, Sousse, Tunisia; ^20^Laboratory of Venoms and Therapeutic Molecules LR11IPT08, Institut Pasteur de Tunis, University of Tunis El Manar, 13 Place Pasteur BP74Belvédère, Tunis Belvédère, Tunisia; ^21^High Institute of Biotechnology of Sidi Thabet, University of Manouba, Ariana BP-66, Manouba 2010, Tunisia

## Abstract

Hackathons are collaborative events that bring together diverse groups to solve predefined challenges. The COVID-19 pandemic caused by SARS-CoV-2 has emphasized the need for portable and reproducible genomics analysis pipelines to study the genetic susceptibility of the human host and investigate human-SARS-CoV-2 protein interactions. To build and strengthen institutional capacities in OMICS data analysis applied to host-pathogen interaction (HPI), the PHINDaccess project organized two hackathons in 2020 and 2021. These hackathons are aimed at developing bioinformatics pipelines related to the SARS-CoV-2 viral genome, its phylodynamic transmission, and the identification of human genome host variants, with a focus on addressing global health challenges, particularly in low- and middle-income countries (LMIC). This paper outlines the preparation, proceedings, and lessons learned from these hackathons, including the challenges faced by participants and our recommendations based on our experience for organizing hackathons in LMIC and beyond.

## 1. Introduction

In December 2019, the very first positive case to SARS-CoV-2 virus, the causative agent of the COVID-19, was identified in Wuhan Province in China. On March the 11th 2020, COVID-19 disease was declared by the World Health Organization (WHO) as a worldwide pandemic. At the writing time (May 2022), there have been more than 520 million confirmed infection cases and 6 million deaths globally. This devastating pandemic has brought together researchers and communities across the globe to tackle this scourge in an unprecedented manner. Indeed, to prevent the transmission of SARS-CoV-2, governments imposed lockdowns on the entire country or in several cities, instructed the public to follow social distancing, and recommended the wearing of masks as primary means of preventing COVID-19 spread [1–3]. High-income countries joined efforts and deployed resources to develop drugs and vaccines against SARS-CoV-2. Soon after the declaration of the pandemic, numerous open access SARS-CoV-2-related databases, including the Global Initiative on Sharing All Influenza Data (GISAID) [4–6], the Coronavirus3D, the National Center for Biotechnology Information [7], the LitCovid, the National Bioinformatics Center (CNCB)/National Genomics Data Center (NGDC) database and the Virus Pathogen Resource, were developed, populated and made public to the scientific community for a rapid variant identification and virus transmission control [8–12]. Similarly, computational tools and resources including the popular genome browser UCSC, the Ensembl database, and the Nextstrain COVID-19 genetic epidemiology were also developed and extended to facilitate SARS-CoV-2 genome assembly, annotation, and variant identification [13–15]. Numerous onsite or online events including workshops, hackathons, and webinar series were organized worldwide to assist countries in COVID-19 case management and efficiently planning their reopenings and national revivals [16, 17].

Among the most popular and reliable sources of solutions to challenges in healthcare that are able to generate enthusiasm for innovation are hackathons [18]. Hackathons have the potential to create an environment where groups of people interrelate their skills, knowledge, and expertise in a friendly, iterative, and collaborative way, making them an ideal solution to rapidly address specific challenges [18, 19]. Likewise, “themed hackathons,” by tackling a specific research subject, offer a unique possibility to swiftly gather experts on a specific subject, enabling them to collaborate effectively in order to solve urgent research matters. As examples, a rehabilitation medicine hackathon that gathered interdisciplinary teams of 102 participants was held in 2015, during which three projects were chosen as prototypes that may improve health conditions for persons with disabilities [20]. Other substantial examples would be the number of hackathons organized in the frame of the COVID-19 pandemic, such as the MIT COVID-19 Challenge initiative in 2020 that emerged in response to the countless challenges posed by the COVID-19 pandemic [21]. Indeed, an extraordinary added value of hackathons is their potential usefulness as a training environment, making a hacking event a niche where new skills are gained, with a very clear domino effect on participants [19].

In our context, the PHINDaccess (Pathogen-Host INteraction Data access) project is a twinning project, coordinated by the Institut Pasteur de Tunis (IPT), that intends to empower its capacities to efficiently exploit the extraordinary potential of omics data generated in the context of pathogen-host interaction (PHI) studies. PHINDaccess has established a genuine partnership with four world-class European institutions evolving at the forefront of infectious disease research and omics science: the Institut Pasteur (IP), the Center for Genomic Regulation (CRG), the Max-Planck-Institute for Molecular Genetics MPI-MG, and the Robert Koch Institute (RKI). PHINDaccess should in fine improve institutional capacities in OMICS data analysis applied to infectious diseases and host-pathogen interactions. In the frame of its main objective, it aims to train and mentor IPT early career researchers that are benefiting from a complete multicomponent training program including both hard and soft skills on PHI.

In the phenomenal context of the COVID-19 pandemic, PHINDaccess project members decided to organize a hackathon on the “Study of genetic susceptibility to COVID-19 and SARS-CoV-2 human host-pathogen interaction” involving project participants. We aimed this hackathon to strengthen IPT OMICS data analysis institutional capacities in pathogen-host interaction and actively participate in international efforts on COVID-19-related data analysis.

This paper discusses the proceedings of the PHINDaccess hackathons, which gathered IPT Tunisian scientists from different backgrounds and various areas of expertise to apply what they have learned in the frame of the PHINDaccess series of training in OMICS data analysis. They designed and developed bioinformatics pipelines and workflows related to SARS-CoV-2 and human public data, producing valuable resources that could be applied to other diseases. We report on our experience running hackathons, highlight key participants' learnings from the hackathon events, explore the various challenges of relevance, and come out with recommendations to run hackathons in an LMIC.

## 2. Materials and Methods

### 2.1. Participants Selection

**Figure 1:**
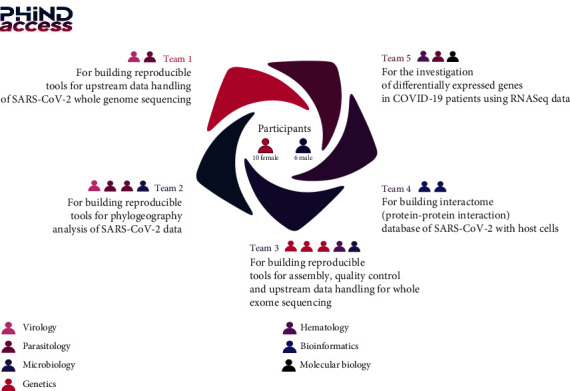
Detailed participants' involvement in the hackathon. The hackathon involved 16 participants from the Institut Pasteur de Tunis. They have been organized in five complementary working groups or “teams.”

PHINDaccess project is a H2020 twinning project aiming to foster and consolidate IPT members' capacities to analyze omics data generated in the context of pathogen-host interaction (PHI) studies. Sixteen participants that undertook the PHINDaccess bioinformatics course series joined the hackathon. Bioinformatics courses that were previously organized and followed by all participants included theoretical and practical sessions, namely, related to “Database development,” “Introduction to Linux,” “Introduction to Python,” “NGS-DNASeq for WGS, WES, and de novo genome sequencing,” “Introduction to R and R studio,” and “NGS-RNASeq for RNA sequencing and data analysis”. These courses were intended to build core staff at the different IPT labs to boost up bioinformatics and computational biology knowledge and skills. Hackathon participants that have previously followed these courses came from different backgrounds as shown in Figure 1. Indeed, the figure shows the number of participants in each team (ranging from 2 to 5 members), the name of the project as well as the background of each participant highlighted by different colors. Within each team, a member has been selected as a leader from the other team members to lead the team and to report back the progress of the project on behalf of the group for the organizers and the other teams. Besides participants, a group of organizers including three bioinformaticians, one system administrator, two project managers, and one member in charge of communication were available all along the event for the logistics, mentorship, for communicating around the event and for ensuring the good progress of the hackathon. The first hackathon event took place from 01 to 09 September 2020, and the second one was held on 28 and 29 October 2021.

### 2.2. Prehackathons Preparation

**Figure 2:**
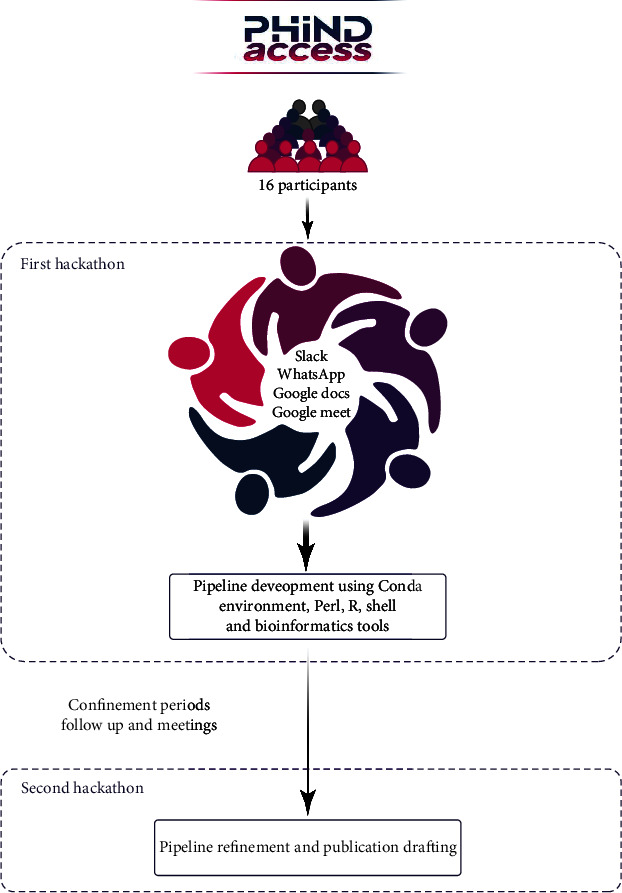
Main hackathon goals and components, including communication platforms.

To better prepare for both events, good and early planning was mandatory to be able to achieve the hackathons expected outcomes and better tackle the challenges. Thus, three months before the first event, monthly meetings were scheduled and occurred through conference calls and face-to-face meetings in order to organize this hackathon. All participants attended these meetings showing their interests and enthusiasm to participate in the events and contribute to make them successful. These meetings allowed us to identify the topic, the time, the duration, the venue, and the format of the hackathon. It also helped to initiate the discussion between all the participants and to identify the specific objectives and potential outcomes. At this level, team members initiated collaborations with each other and started to identify data sources and types of data to be collected as well as the list of tools that were needed, which facilitated the work at the start of the first hackathon. Numerous collaborative tools for centralizing teams' communication and material sharing were employed including a Slack channel and a WhatsApp group. Google Meet was used to conduct meetings. Google Docs was utilized for sharing minutes of meetings and centralizing documents.  Figure 2 summarizes the main goals and components of the hackathons, highlighting the communication platforms.

**Figure 3:**
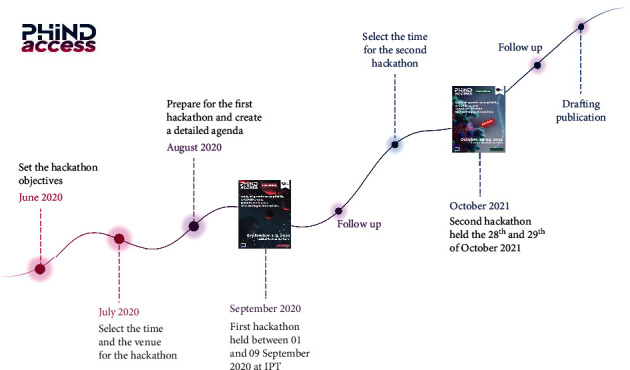
A timeline of the hackathons' planning activities.

Among the participants, few displayed strong skills in omics data analysis and were already familiar with bioinformatics tools, pipeline development, and Linux basic commands. Based on their willingness, background, and research areas, the hackathon's participants were grouped into five teams (Figure 3). Team 1 for building reproducible tools for upstream data handling of SARS-CoV-2 whole genome sequencing, team 2 for building reproducible tools for phylogeography analysis of SARS-CoV-2 data, team 3 for building reproducible tools for assembly, quality control, and upstream data handling for whole exome sequencing, team 4 for building interactome (protein-protein interaction) database of SARS-CoV-2 with host cells, and team 5 for the investigation of differentially expressed genes in COVID-19 patients using RNASeq data. The diversity and the complementary areas of expertise within the teams created a perfect work environment enabling hackathon goals achievement. Based on the repartition of teams, a centralized official GitHub repository was created. The five subprojects were tracked down from their personal repositories and were merged within the centralized main one in an effort to make the project's organization and access clearer and easier.

### 2.3. Tools Installation and Bioinformatics Environment Preparation for the Hackathon

Bioinformatics tools are increasingly used in the field of modern biology in order to organize, analyze, visualize, and correctly interpret biological data. To better prepare the proceedings of the hackathons, a series of basic and advanced bioinformatics tools running on the Ubuntu 20.04 operating system were installed and configured by the system administrator using automated scripts before the hackathon venue. Furthermore, all participants have been previously initiated to the use of the Conda environment to create, export, list, remove, and update environments that have different versions of Python and/or packages installed in them. Participants could therefore activate, deactivate, and switch between environments when needed to use the appropriate tools useful for their analyses. The major advantage is that they were able to list all the created environments and easily switch between environments by activating and deactivating the laters. Users were also able to share the created environment with a team member using an environment.yml file to recreate the same environment with all needed tools ensuring the reproducibility of computational pipelines. Using such command lines, participants were able to apply in practice what they learned during previous courses organized in the frame of the PHINDaccess project. Participants showed confidence with the command-line tool to explore the operating file system, create files, and move between directories. They were able to use Conda to manage the computational environment and load appropriate bioinformatics tools used for these hackathons. Finally, they familiarized themselves with their personal GitHub repositories.

### 2.4. Hackathons Proceedings and Activities

#### 2.4.1. First Hackathon

The first hackathon was organized in IPT from 01 September to 09 September 2020, corresponding to seven working days.

In developing pipelines, participants were expected to test, optimize and benchmark different bioinformatics tools. Often, these activities need to be performed on different computer hardware that is not necessarily compatible or on operating systems to which users do not possess full control or lack expertise that allows them to compile and install the required tools. For such a reason, participants were trained on how to use the Conda virtual environment with a special focus on the capabilities offered by the bioconda channel that allows access to more than 9000 bioinformatics packages that could be integrated into workflow management tools. Practical tutorials aiming to teach participants how to create virtual environments, how to install specific versions of software, and how to reproduce and share the environment's components using yaml files were therefore set up during this session.

Bioinformatics projects may become complicated by different interfering tasks and nonlinear development as well as the diversity of data and the involvement of multiple contributors. Some of the problems that can arise from a complex project are the creation of conflicting versions by collaborators and the difficulty to spot bugs and fixing errors in workflows. For such reasons, participants undertook practical training on the git version control system. The session focused on topics regarding how to create repositories, how to initiate and monitor the tracking using git, and how to manage remote repositories and conflicting git commits. The hackathon program was based on a series of presentations and talks highlighting the objectives of the different projects. The first day of the hackathon was dedicated to a detailed presentation of the objectives, the format, and the composition of the teams followed by a discussion concerning the planning of the week. For the following days, teams split into their individual collaboration areas at IPT to plan their strategies, identify the data to be collected, and select the tools to be used. A report-back session was scheduled at the end of each day, during which each team leader presented the procedures followed, the advances made, and the challenges that have been faced. Because they were made by each team in front of the others, these wrap-up sessions helped each team to receive valuable comments from all team members and created a room for beneficial panel discussions. This helped teams to clarify views, consolidate efforts, rectify procedures, and fine-tune strategies. To efficiently organize each working day, a day-to-day detailed working plan was prepared at the end of the day before. The panel discussions also presented the progress made in the frame of the working plan, which helped refine each daily program and prevent delays. For the three last days of the hackathon, participants were obliged to switch from face-to-face meeting to online meetings due to the first COVID-19 wave and the sudden increase in the number of COVID-19 positive cases among IPT personnel. Although the five different teams achieved significant progress toward data collection, appropriate tool identification, installation, and testing during the hackathon, further work was required for all the projects. Thus, the team members committed to contributing and investing time to finalize the development of their bioinformatics pipelines in the frame of the five projects.

#### 2.4.2. Interhackathon Work

At the end of the first hackathon, milestones and task assignments were set by members of each team/project. These tasks consisted of steps needed to finalize the development and the running of the distinct pipelines. The progress of these tasks was supervised by constant meetings between the project members through platforms such as Slack, WhatsApp, and Google Drive. A series of meetings with mentors and organizers were convened to monitor and gauge the progress as well as the team productivity in solidifying and achieving the pipeline development between both hackathon events.

#### 2.4.3. Second Hackathon

A second hackathon was scheduled during these meetings to refine the developed pipelines and valorize the work done by the teams. The second hackathon took place on the 28th and 29th of October 2021, in which members of the five projects met again for two days to polish the developed pipelines and start to draft potential publications. A summary of the hackathons' timeline planning is provided in Figure 3.

### 2.5. Data Sources and Types

The first working group is aimed at validating and setting up a pipeline for SARS-CoV-2 variant study and SNP identification. For this, they downloaded a sample SARS-CoV-2 genome (SRR12532546) from GenBank on 03 September 2020. This sample was isolated from the Philippines, and its data were generated using the paired-end library on the Illumina platform. In addition, the team used the sequence NC-045512.2 of the established severe acute respiratory syndrome coronavirus 2 reference sequence isolated from Wuhan-Hu-1. The main goal of the second team was to understand the early transmission dynamics of the SARS-CoV-2 in Africa. To reach their objective, whole genome FASTQ sequences related to 1800 SARS-CoV-2 isolates from Africa, and references from all over the world were downloaded from the GISAID database [4–6]. Data was downloaded with information regarding Nextstrain type, clade, collection's date, and location. The sequences were selected to be representative of all African countries in a balanced way. Team 3 is aimed at developing a reproducible pipeline for joint analysis of multiple human whole exome sequences (WES). The team initiated their work by extracting human exome data sets from the EMBL-EBI data resources (FASTQ files) [22]. Team 4 is aimed at developing a database of human-SARS-CoV-2 protein-protein interactions collected from available databases and curated from the literature. In brief, the PubMed database was consulted using appropriate keywords to retrieve all publications related to human host-SARS-CoV-2 interactions. Finally, team 5 performed an RNAseq analysis approach to investigate differentially expressed genes in COVID (+) patients compared to COVID (-) control patients by means of distinct bioinformatics tools and pipelines.

## 3. Results and Discussion

Team 1, which involved two participants, was able to develop a full pipeline, allowing the assembly of the SARS-CoV-2 viral genome, as well as the identification of all mutations within the genome by comparison to the NC-045512.2 reference sequence isolated from Wuhan-Hu-1. Briefly, the team initiated its work by trimming adaptor sequences and primers from the SRR12532546 sample using the Trimmomatic 0.33 tool. Quality control of data was performed using the FastQC software [23]. The cutoff of read depth was set to 30. Good sequences (*Q*-score >30) were firstly mapped into the human genome using the BWA tool. Only unmapped reads were assembled to contiguous sequences (contigs) using the SPAdes algorithm software [24]. The team processed only high-quality reads having at least 80% of base pairs with base-calling accuracy of 99.9% and used stringent assembly parameters in the SPAdes assembler. The ABACAS algorithm was used for contig ordering and genome draft generation [25]. To identify mutations, the obtained contigs were firstly clustered at 3% pairwise distance, and a consensus sequence was generated. Consensus sequences were used to extract the mutation points from each genome including substitutions, deletions, and insertions.

Team 2, focusing on the study of the transmission dynamics of the SARS-CoV-2 and its introduction in Africa, collected complete genome sequences related to 1800 SARS-CoV-2 isolates from Africa and references from all over the world. Bayesian coalescent analyses were performed on major lineages of the Nextstrain build in which (number of sequences of isolates from Africa as well as references from all over the world) sequences fell. The purpose of these analyses was to (i) confirm the estimated date of origin for SARS-CoV-2, (ii) infer the estimated date to the most recent common ancestor for major lineages, and (iii) infer the estimated dates of viral introductions into Africa. The full pipeline is available within the GitHub repository.

The COVID-19 pandemic has accounted for millions of infections and hundreds of thousand deaths worldwide. Patients demonstrated a great diversity in clinical manifestations and disease severity. However, little is known about the host genetic contribution to the observed interindividual phenotypic variability mainly in African populations.

**Figure 4:**
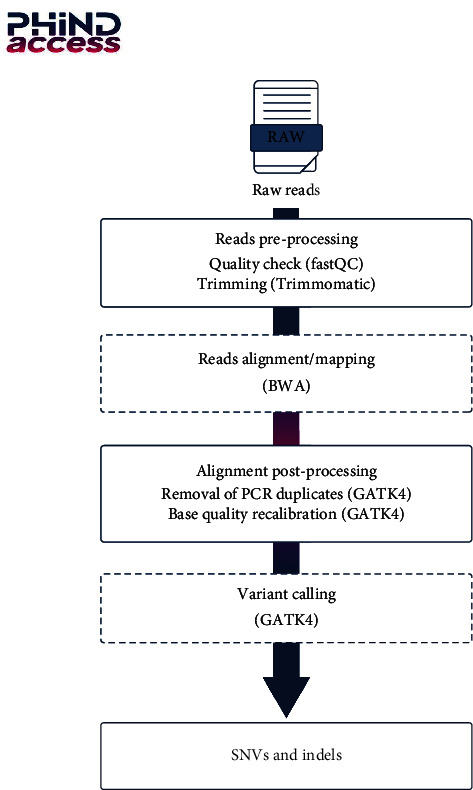
Example of a workflow for WES data analysis pipeline.

Whole genome sequencing (WGS) and whole exome sequencing (WES) methods were used to detect these genomic variants. While WGS is more and more used in research and clinical diagnosis of hereditary diseases, WES is a more economic approach compared to WGS and is becoming a standard. The aim of team 3 was to develop a reproducible pipeline for the simultaneous analysis of multiple human whole exome sequences (WES) that could be used for host genetic studies and analysis. Until recently, most WES pipelines used for genetic variant discovery process one sample at a time. During this hackathon, team 3 endeavored to develop a reproducible pipeline for joint analysis of multiple human whole exome sequences (WES). To do so, the team employed many “for” loops all along the pipeline, in order to iterate the different analysis steps for multiple samples. The pipeline displayed in Figure 4 covered all parts of the whole exome sequencing workflow including quality control processing of raw data, alignment phase, postalignment processing, and variant calling and filtration. Importantly, the team integrated the GATK most updated version, GATK4, to benefit from its highest efficiency. The pipeline is shared in GitHub and could be used for host genetic studies and analysis.

Using a combination of keywords including “SARS-CoV-2 human proteins interactome” on the PubMed database, team 4 obtained 126 results. The team proceeded with a first manual curation of abstracts to select articles that provided a list of SARS-CoV-2 human protein-protein interactions. The selected articles were then retrieved, and a second manual curation on the whole content of papers was performed by team members to retrieve all viral-host protein interactions. In total, 2989 interactions involving 33 SARS-CoV-2 proteins and 1483 human proteins were retrieved. Based on the collected data, an R script using R shiny, visNetwork, and shiny dashboard packages was built to retrieve data from generated csv files and display it on the web interface over the web. An in-house Perl script was developed to make protein interactions in appropriate format. All data and scripts are available on GitHub.

In order to investigate differentially expressed genes in COVID patients using the RNAseq analysis approach, a set of data was chosen from the National Center for Biotechnology Information Search database (NCBI) by team 5. The aim was then resumed in a comparison of the expressed genes in COVID(+) vs. COVID(-) samples and the identification of the over- and underexpressed genes by the means of different tools. To be able to achieve their goals, members of the Team 5 designed a bioinformatics pipeline starting by the quality control where they compared outputs generated by two softwares FasTQC and RSeQC. The second step was the trimming of low-quality data and adapters using Trimmomatic and Skewer. The third step corresponds to the alignment of the retained reads against the reference genome with BWA, STAR, and SALMON [26–28]. Finally, the identification of the differentially expressed genes between COVID(+) patients and COVID(-) individuals has been performed with DESeq2 and edgeR [29].  Table 1 lists all the tools and databases used in the frame of both hackathons. Despite the efforts and the hard work, the team faced some challenges that will be reported in the next section.

### 3.1. Challenges of the Hackathons

One of the major aims of this hackathon was to give to participants the opportunity to apply what they learned from the PHINDaccess course series and put into practice the acquired knowledge. While some teams succeeded to reach their initial goals, other teams were confronted with many issues to reach their aims. In the following, we report challenges raised by participants during the hackathons.

Regarding the communication between team members, due to the COVID-19 pandemic and the series of successive confinement periods, it proved difficult to ensure regular meetings between team members. Besides these challenges, the team members faced some technical challenges, mainly related to the internet connection. In fact, IPT, as every structure in an LMIC, has a relatively poor quality internet connection, summed up in two points: a low internet bandwidth and an unstable internet connection leading to frequent loss of the connection. This low bandwidth can either be caused by the Mbps rate that the current broadband has, or the bandwidth could be slowed down because too many people are connected to the network at the same time. During the hackathon, slow internet connection has prevented some team members from downloading real OMICS raw data (several gigabytes per file) from a public biorepository in order to be able to develop their analysis pipelines, leaving them with a feeling of frustration. Some teams decided finally to retrieve dummy data (several megabytes per file) to overcome this issue. Another encountered challenge consisted in the lack of intensive computation resources available at the time of the hackathons. It is well known that a comprehensive analysis of omics data requires vast computational resources for multistep analysis pipeline development. These computational resources can be summarized in the number of available CPUs and the amount of memory available on the machine. Again, some team members dealing with a large quantity of data faced these challenges.  Table 2 summarizes the challenges and troubleshooting encountered during both events.

### 3.2. Hackathons Feedback and Lessons Learned

**Figure 5:**
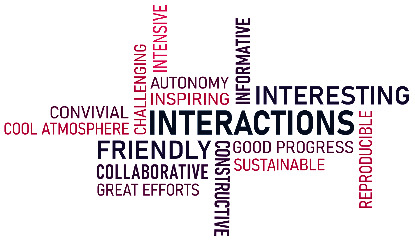
The hackathons as seen by participants.

After the hackathon, the participants were requested to provide assessments on what they thought of the event, how they found the atmosphere and the process, what they learned during the first hackathon, what they learned after both hackathons, what was the outcome of the two hackathons on their works, and what are the challenges faced during both hackathons. The organizers came up with a survey consisting of a few simple questions and, in the following, the participants' feedback. All participants responded to the survey. Link to the survey is provided at the end of this manuscript. Regarding what they learned during the first hackathon, four answers out of 16 included team and collaborative work. In addition, participants mentioned that they learned about the utility of the Conda package management system and applied what they learned during the PHINDaccess course series. Throughout these hackathons, they were able to develop bash and R scripts calling appropriate bioinformatics tools for their respective projects and interact with the GitHub repository to make their scripts public. Participants appreciated the hackathon topic, the organization, the collaborative work, and the interactions between group members. Concerning what they learned after both hackathons, answers included the way to work remotely due to the COVID-19 pandemic and the ability to develop some pipelines using bioinformatics tools integrated within a Conda environment. Concerning what participants appreciated the most during the hackathon, nine participants highlighted the familial and friendly atmosphere, the teamwork and the diversity of team members and their expertise. Participants were also asked if they were able to run or organize such events on their own. Eight participants responded that they were able to organize such events on their own. 12 participants were able to apply what they learned in the frame of the series of omics training performed in the frame of the project including Linux commands, R language, Python, and the NGS data quality control and trimming. The latest answers highlighted the utility of the series of courses followed by the trainees before the organization of both hackathons. While the objectives of both hackathons consisted to strengthen the capacity of IPT users in OMICS data analysis, some teams seem to be more ready to publish their work than other teams who encountered some challenges including internet connection and a delay in public raw data download. Gathering these feedbacks was highly useful for the hackathon organizers to understand how the events were perceived by the participants and how to better plan for next similar events. Finally, Figure 5 highlights the words mostly used by the participants to describe both events.

### 3.3. Hackathons Limitations

Although both events were deemed successful, we identified some limitations that were noticed by the participants and/or the organizers that could raise several constraints and pose a set of hurdles affecting the scope and outcomes.

For instance, the relatively smaller number of participants in this event compared to other ones organized in more developed locations could have significant drawbacks. The participation of a smaller number of persons with the necessary set of skills could affect the diversity of ideas and viewpoints brought to the hackathon, potentially limiting the solutions developed.

Another aspect that can highly limit the outputs of these events is the technical issues related to internet traffic. Due to this and the fact that the needed data were very large, the participants' ability to download and analyze these big multiomics datasets was greatly hampered. These limitations may have a significant impact on the quality of the hackathon outcomes.

### 3.4. Recommendations for LMIC and Concluding Remarks

IPT is an academic institution based in a LMIC. In the present section, we will summarize important organizational and technical recommendations that we believe are essential for organizing a successful hackathon within LMIC settings. These recommendations are derived from valuable insights gained during the conducted hackathon and curated after a comprehensive analysis of the hackathon experience and the subsequent identification of critical gaps.

#### 3.4.1. Organizational Recommendations

In order to organize a successful hackathon, organizers should firstly decide the theme and define the goals of the scheduled hackathon as well as the target audience at least six months ahead of time. Organizers have to identify the skills and profiles of the participants needed to be able to achieve the goals of the hackathon. This will help define a solid general guideline for the event and ensure that the participants have the necessary skills and knowledge to run the hackathon successfully.

Secondly, organizers have to decide the hackathon format (offline, online, and hybrid), the timing (how many days?), the date, the venue, the sponsors, if any, and need to set up rules or a code of conduct during and after the hackathon. Organizers should avoid public holidays, weekends, and religious days when choosing the date of the hackathon. Despite the fact that this may prove to be somewhat restrictive for the organizers and participants in terms of their personal schedules, it will guarantee that the majority of participants can attend and be present throughout the process.

Thirdly, organizers need to set up the program of the hackathon, the list of speakers that will be involved, and the moderators of each session. If speakers are involved, they should be contacted to have their approval for participation. These steps need to be dealt with around two to three months before the hackathon. One month before the event, organizers should contact and sign on food caterers and miscellaneous vendors such as T-shirt suppliers. During the hackathon, organizers should ensure the good progress of the registration, the respect of the time dedicated to each session within the program, and the well-being of participants during the whole period of the hackathon. These recommendations are of the utmost importance in ensuring the smooth progress of the different steps involved during the hackathon. However, this absolutely requires a high level of communication between the organizing team. They also need to communicate on the event by generating videos and photos of the event for marketing purposes, as media coverage could provide important visibility for the event and ensure future collaborations.

It is also recommended to establish a contingency plan when organizing an “in-person hackathon”. For instance, in our scenario when confronted with the COVID-19 pandemic, the organizers swiftly transitioned from an “in-person hackathon” to a virtual/hybrid format by having a well-thought-out alternative plan in place, thereby maintaining the integrity of the event and minimizing disruptions to participant engagement, ensuring safety, and maintaining collaboration. This proactive approach, if unforeseen circumstances arise, safeguards the hackathon's objectives and outcomes while demonstrating an adaptive and resilient organizational strategy. It is very important to share surveys and forms with participants to get their feedback regarding the event proceeding, the experience gained, and what has been learned. In that sense, a pre- and posthackathon survey would allow organizers to gauge improvements from the hackathon. Finally, a follow-up should be performed by the organizers after the event to ensure that all fixed objectives are fulfilled.

#### 3.4.2. Technical Recommendations

One of the major issues in LMIC institutions is the internet connection where the bandwidth is low, the internet is not stable, and the costs tend to be the highest. Hackathon organizers should ensure an acceptable internet connection to allow participants to retrieve data from public databases, to consult the literature and web sites, to communicate with each other, to exchange documents, scripts, and pipelines, and to work in a collaborative way. During our first hackathon, we faced some internet issues, and one group working on expression data analysis spent a lot of time downloading RNASeq data from the SRA repository as many users have been using the network at the same time. Thus, for huge volumes of data, we recommend organizers to prepare and download the dataset for participants in advance before the event and make it available for participants at the starting of the event to avoid spending time downloading data during the event. Furthermore, to be able to analyze OMICS data, organizers should ensure the availability of computational resources and memory as bioinformatics pipelines require minimum resources. Finally, in case of having a stable internet connection but a lack of appropriate computational resources for hackathon purposes, cloud computing can serve as a viable alternative as the latter allows a faster deployment to get the resources required at low cost with all softwares integrated without any additional efforts to integrate applications.

## 4. Conclusions

In conclusion, the present paper reports on the PHINDAccess consortium experience to organize and run omics data analysis hackathons related to COVID-19 host-pathogen interaction prediction. It highlights all the organizational steps undertaken, details the hackathon proceedings, reports key participants' learnings from the events, explores the various challenges of relevance, and comes out with recommendations to run hackathons in LMICs.

## Figures and Tables

**Table 1:** Summary of tools, databases and repository used during the hackathons.

Databases		GenBank (https://www.ncbi.nlm.nih.gov/genbank)
		GISAID (https://gisaid.org/)
		EBI/ENA (https://www.ebi.ac.uk/ena/browser/home)
		PubMed (https://pubmed.ncbi.nlm.nih.gov/)
		NCBI (https://www.ncbi.nlm.nih.gov/)

Tools	Communication tools	Slack
		WhatsApp
		Google Meet
		Google Docs

Tools	Bioinformatics tools	FastQC
		Trimmomatic
		BWA
		SPAdes
		GATK4
		Shiny, ShinyDashboard, visNetwork
		RSeQC
		Skewer
		STAR
		SALMON
		DESeq2
		EdgeR

	Programming language	Perl
		R
		Shell
		Python
		Nextflow

Repository		GitHuB

**Table 2:** Overview of the obstacles faced and problem-solving methods used during the hackathon.

Hackathon challenges	Communication challenges	Difficulty of setting up meetings between team members on a regular basis because of the COVID-19 pandemic and confinement.

Hackathon challenges	Technical challenges	Poor quality of the internet connection due to both limited bandwidth and an unstable connection
		Difficulty to download OMICS raw data from a public biorepository.
		Lack of intensive computation resources available at the time of the hackathon.

Troubleshooting		Organizing regular online meetings through communication platforms.
		To overcome the downloading issue, some teams ultimately opted to retrieve dummy data.

## Data Availability

Data are available at https://github.com/hothman/PhindAccessHackathon2020. A link to the survey is provided at https://docs.google.com/forms/d/e/1FAIpQLSfuv3jfk2Hq27XrTHE2MjmRTDO957M46CS75AHBQto-nRUWnw/viewform.
